# Bile Culture, Antimicrobial Susceptibility, and Hepatobiliary Pathology in Dogs Undergoing Cholecystectomy for Gallbladder Mucocele

**DOI:** 10.3390/ani16010031

**Published:** 2025-12-22

**Authors:** Ji-Min Choe, Hyoju Kim, Jeonyeon Hwang, Hwi-Yool Kim

**Affiliations:** 1 Helix Animal Medical Center, Seoul 06222, Republic of Korea; cjm59@naver.com (J.-M.C.);; 2 Department of Veterinary Surgery, College of Veterinary Medicine, Konkuk University, Seoul 05029, Republic of Korea

**Keywords:** dog, cholecystectomy, bile culture, antimicrobial resistance, gallbladder mucocele, liver biopsy, histopathology

## Abstract

Gallbladder mucocele is a major cause of biliary disease in dogs, particularly affecting elderly and small-breed populations. Background: This study retrospectively evaluated 65 dogs that underwent cholecystectomy at a referral hospital in Seoul, Korea, (2022–2025) to characterize clinical features, bile culture results, antimicrobial susceptibility, and histopathologic findings. Methods: Signalment, ASA grade, preoperative laboratory data, bile culture and susceptibility results, and postoperative outcomes were reviewed. Results: Bile culture was positive in 21.3% (13/61) of cases, most frequently isolating *Escherichia coli* 38.5% (5/13) and *Enterococcus* spp. 30.8% (4/13). Approximately 18% of isolates were multidrug- or extensively drug-resistant, showing reduced susceptibility to β-lactams but retained sensitivity to imipenem and florfenicol. Preoperative increases in liver enzyme activities and C-reactive protein were common. Histopathology revealed gallbladder mucosal hyperplasia 90.4% (47/52) and inflammation 61.5% (35/52), often accompanied by hepatic inflammation or fibrosis. Toy Poodles were significantly over-represented (35.4%; OR = 3.1, *p* < 0.001). Antibiotic matching was not significantly associated with complications or the length of hospital stay (LOS). Conclusions: Although the bile-culture positivity rate was modest, the frequent detection of multidrug-resistant (MDR) and extensively drug-resistant (XDR) organisms highlights the clinical value of culture-guided antibiotic therapy. Routine bile culture with susceptibility testing, concurrent liver biopsy, and careful perioperative management may improve diagnostic accuracy and outcomes in canine gallbladder disease.

## Simple Summary

Gallbladder mucocele is increasingly recognized in small-breed dogs. This retrospective study analyzed 65 dogs that underwent cholecystectomy at a veterinary referral hospital in Seoul, Korea, between 2022 and 2025. Toy Poodles were the most frequently affected breed. Preoperative abnormalities in liver enzyme activities and elevated C-reactive protein levels were common. Bile culture was positive in 21.3% of cases, and approximately 18% of isolates showed multidrug- or extensively drug-resistant patterns, most often *Escherichia coli* and *Enterococcus* spp. Although culture positivity was modest, clinically important antimicrobial resistance was identified. These findings highlight the value of bile culture and susceptibility testing in dogs with gallbladder disease and support the use of concurrent liver biopsy and careful perioperative management to enhance diagnostic assessment and guide individualized clinical decision-making.

## 1. Introduction

Gallbladder disease is a significant cause of morbidity and mortality in dogs, particularly among geriatric and small-breed populations [1]. Gallbladder mucocele (GBM), one of the most common biliary disorders requiring cholecystectomy, has shown increasing prevalence in recent years [2]. Affected dogs typically present with nonspecific signs such as anorexia, vomiting, lethargy, abdominal pain, and jaundice, accompanied by elevations in hepatobiliary enzymes and characteristic ultrasonographic abnormalities [3]. Without timely surgical intervention, GBM may progress to biliary obstruction or gallbladder rupture, potentially resulting in septic peritonitis and death [4,5].

Therefore, cholecystectomy remains the recommended treatment for clinically significant disease [6]. Despite advances in surgical and anesthetic management [7,8], postoperative outcomes still vary widely [9], likely reflecting the multifactorial nature of GBM, where gallbladder pathology, systemic inflammation, hepatic comorbidities, and infectious components interact. GBM development is currently understood to involve impaired gallbladder motility, biliary stasis, and dysregulated mucin secretion, leading to progressive inspissation and intraluminal organization of bile [10]. Metabolic and endocrine disorders such as hyperadrenocorticism, hypothyroidism, and hyperlipidemia have been proposed as contributing risk factors [2,11], and chronic systemic inflammation may further intensify epithelial injury [4,5]. In addition, several small-breed dogs appear disproportionately affected, suggesting a potential genetic predisposition in certain populations [2,3,12,13]. These mechanisms underscore the multifactorial nature of GBM and support comprehensive evaluation beyond gallbladder-limited pathology [10,14]. Bacterial infection within the biliary tract is recognized as an important contributor in a subset of dogs [15,16]. Escherichia coli and Enterococcus spp. are frequently isolated from affected patients, suggesting an enteric origin of infection [17,18]. However, reported bile culture positivity varies across studies [15], and the prognostic implications remain unclear [18].

Meanwhile, multidrug-resistant (MDR) and extensively drug-resistant (XDR) organisms are increasingly detected in bile samples [18], emphasizing the risk of empirical β-lactam therapy [19]. The liver and gallbladder are closely interconnected in health and disease. Histopathology often reveals mucosal hyperplasia, chronic inflammation, and fibrosis in the gallbladder, with concurrent hepatic lesions commonly observed [10,14]. Increasing evidence suggests that GBM reflects systemic hepatobiliary involvement rather than gallbladder-limited pathology [10]. However, liver biopsy is not routinely performed in all cases, and comprehensive evaluation remains limited. Breed predisposition is another important epidemiologic factor, with several small breeds—such as Shetland Sheepdogs, Cocker Spaniels, and Poodles—reported to be at high risk [2,12,20].

Genetic factors including an ABCB4 insertion mutation have been implicated in biliary pathology [21]. Recent studies in Korea further emphasize Toy Poodles as a high-risk breed, highlighting the importance of regional data [3]. Although awareness of GBM has improved, critical aspects remain insufficiently characterized. Few studies have simultaneously assessed bile microbiology, antimicrobial resistance, hepatobiliary histopathology, and postoperative outcomes in dogs undergoing cholecystectomy [4].

Additionally, data from Korean veterinary hospitals remain scarce, limiting evidence-based prediction and standardized perioperative management. Therefore, this retrospective study aimed to (1) investigate the prevalence and clinical significance of bile culture positivity in dogs undergoing cholecystectomy, (2) characterize antimicrobial susceptibility profiles of biliary isolates, including MDR/XDR phenotypes, (3) assess gallbladder and hepatic histopathology, and (4) analyze breed distribution—with a focus on Toy Poodles—to identify potential predisposition patterns in a Korean cohort.

By integrating microbiologic, clinicopathologic, histopathologic, and epidemiologic findings, this study aims to help guide clinical decision-making and improve outcomes in dogs undergoing cholecystectomy.

## 2. Materials and Methods

### 2.1. Study Design and Case Selection

This study was performed at Helix Animal Medical Center, Seoul, Republic of Korea, and included client-owned dogs that underwent cholecystectomy between January 2022 and September 2025. Surgical indications were based on ultrasonographic and clinicopathologic findings consistent with gallbladder disease. All procedures were based on previously described protocols [6,22] using a standard open approach under general anesthesia, with routine removal of the gallbladder following ligation of the cystic duct and artery. All dogs received postoperative broad-spectrum antimicrobial therapy regardless of bile culture status or antimicrobial susceptibility results.

All data collection and follow-up were censored at 10 September 2025. Medical records were reviewed to collect information on signalment, ASA physical status classification, comorbidities, preoperative laboratory results, ultrasonographic findings, bile culture and antimicrobial susceptibility results, histopathologic findings of the gallbladder and liver, and postoperative outcomes. The ASA physical status classification was used to assess the anesthetic risk of each patient, following the American Society of Anesthesiologists’ system (I–V) [23].

Only cases with a cholecystectomy procedure and at least 70% of perioperative clinical data available were included in the analysis. Cases were excluded if the surgery was aborted before completion, if the same patient underwent repeat surgery (in which case only the initial procedure was retained), or when essential information—such as the date of surgery, histopathological findings, or postoperative outcomes—was absent from the medical record.

### 2.2. Data Collection and Variables

The following data were extracted from medical records: patient characteristics (age, sex, body weight, breed, and ASA physical status), comorbidities (systemic diseases that could influence anesthetic or prognostic risk, such as diabetes mellitus, hyperadrenocorticism, chronic kidney disease, cardiac disease, hepatic disease, or systemic neoplasia), and preoperative laboratory parameters including ALT, ALP, AST, GGT, total bilirubin, albumin, white blood cell count, and C-reactive protein (CRP). Serum biochemical analyses were performed using an automated dry chemistry analyzer (DRI-CHEM NX700; Fujifilm, Tokyo, Japan) and a wet chemistry analyzer (BS-430; Mindray, Shenzhen, China). Hematological parameters were measured using an automated hematology analyzer (BC-60R Vet; Mindray, Shenzhen, China). Reference intervals for serum biochemistry and hematology were based on the manufacturer-provided ranges for canine samples established by each analyzer.

### 2.3. Imaging Evaluation

Abdominal ultrasonography findings were reviewed and classified according to previously described criteria. Gallbladder content was categorized into four patterns based on echogenic characteristics: Pattern I (immobile sludge), Pattern II (stellate or striated echogenic bile), and Patterns III–IV (classic “kiwi fruit” or complex/organized pattern). A diagnosis of gallbladder mucocele was made when immobile echogenic material with any of these characteristic patterns was identified within the gallbladder lumen [3]. Biliary obstruction was recorded as present or absent based on ultrasonographic evidence of extrahepatic biliary duct dilation or impaired bile outflow, consistent with previously described diagnostic criteria.

### 2.4. Microbiologic Analysis

Bile samples were aseptically collected postoperatively via cholecystocentesis. Both aerobic and anaerobic bacterial cultures were performed by accredited laboratories, GREENVET, Yongin, Republic of Korea and KVL, Seongnam, Republic of Korea. Aerobic isolates were identified using matrix-assisted laser desorption/ionization time-of-flight mass spectrometry (MALDI-TOF MS), biochemical tests, and Gram staining. Antimicrobial susceptibility testing (AST) was performed using the broth microdilution method to determine minimum inhibitory concentrations (MICs) and the disk diffusion method to evaluate zone diameters according to Clinical and Laboratory Standards Institute (CLSI) [24] and European Committee on Antimicrobial Susceptibility Testing (EUCAST) [25] guidelines. If species-specific breakpoints were unavailable, surrogate interpretive criteria for *Escherichia coli* (Gram-negative) or *Staphylococcus aureus* (Gram-positive) were applied. Anaerobic cultures were performed in a limited number of cases; however, they were not included in the final analysis because the data was incomplete and standardized AST criteria are lacking. Methicillin resistance in *Staphylococcus* spp. was confirmed using oxacillin or cefoxitin testing. *Enterococcus* spp. isolates were interpreted as intrinsically resistant to cephalosporins, clindamycin, and trimethoprim–sulfamethoxazole. Culture results were classified as negative when no bacterial colonies were observed within 48 h.

### 2.5. Antimicrobial Resistance Classification

Resistance profiles were categorized according to previously described criteria [26]: Pan-susceptible (Pan-S, susceptible to all tested antibiotics), multidrug-resistant (MDR, resistant to ≥3 antimicrobial classes), extensively drug-resistant (XDR, susceptible to ≤2 classes), and pan-drug resistant (PDR, resistant to all tested antimicrobials, none identified). Antibiotic matching was defined by comparing perioperatively administered antibiotics with AST results: if ≥1 antibiotic was susceptible, coded as match; if all were resistant/intermediate, coded as mismatch.

### 2.6. Postoperative Outcomes and Definitions

Postoperative clinical outcomes were evaluated using standardized criteria. Major complications were defined as postoperative events requiring surgical, procedural, or advanced medical intervention, including septic peritonitis, bile leakage, hemodynamic instability requiring vasopressors, significant hemorrhage, or respiratory failure requiring oxygen supplementation or mechanical support. 30-day mortality was defined as death occurring within 30 days after surgery, regardless of cause. Postoperative intensive care was recorded when continuous monitoring, oxygen therapy, vasopressor support, transfusion, or other advanced medical interventions were required during hospitalization. Readmission was defined as any unplanned hospitalization occurring within 90 days of discharge. These variables were extracted directly from the electronic medical record system and analyzed as described in the statistical analysis section.

### 2.7. Histopathological Evaluation

Gallbladder and liver samples were submitted to two commercial veterinary pathology laboratories (KVL and Greenvet). Liver biopsy samples were obtained intraoperatively in most cases using either punch biopsy or the guillotine ligation technique, depending on the anatomical accessibility and the surgeon’s preference. The biopsy was performed on grossly abnormal areas when present, or on the left lateral hepatic lobe when no focal lesion was visible.

All tissues were fixed in 10% neutral-buffered formalin for a minimum of 24–48 h, routinely processed, and embedded in paraffin. Sections were cut at 3–4 μm thickness and stained with hematoxylin and eosin (H&E) according to each laboratory’s standard protocol.

Gallbladder scoring included inflammation (none, mild, moderate, severe), fibrosis (none, mild, moderate, severe), mucosal hyperplasia (present/absent), and perforation (present/absent). Liver scoring included hepatitis grade (0–3), cholangiopathy (absent/mild/moderate/severe), fibrosis (absent/mild/moderate/severe). Histopathological grading followed previously established veterinary pathology protocols [10]. The type of inflammatory infiltrate (neutrophilic, lymphocytic, or mixed) could not be systematically classified across liver and gallbladder samples because most pathology reports did not specify the predominant inflammatory cell type.

All histopathological evaluations were performed by a board-certified veterinary pathologist who was blinded to clinical information and microbiologic findings.

### 2.8. Statistical Analysis

All analyses were performed using IBM SPSS Statistics version 29.0 (IBM Corp., Armonk, NY, USA). Normality of continuous variables was evaluated using the Shapiro–Wilk test. Continuous data were summarized as mean ± standard deviation (SD) or median (range), and categorical variables as frequencies (%). The binomial test was applied to estimate bile culture positivity against a 5% baseline rate and to determine the proportions of MDR/XDR isolates and breed overrepresentation. Comparisons between categorical variables, including complication occurrence and antibiotic matching versus clinical outcomes, were analyzed using the Chi-square or Fisher’s exact test. The association between antibiotic matching and the length of hospital stay (LOS) was evaluated using a negative binomial regression model for count data. Overdispersion was tested using the Lagrange multiplier test (*p* > 0.05). Regression coefficients (B) were exponentiated to obtain rate ratios (Exp(B)) with 95% confidence intervals (CIs). Model fit was assessed using Pearson χ^2^/df and Deviance/df values. Statistical methods and interpretations followed standard biostatistical principles described by [27,28]. Statistical significance was set at *p* < 0.05.

## 3. Results

### 3.1. Baseline Characteristics of Dogs Undergoing Cholecystectomy

During the study period, 65 dogs underwent cholecystectomy. The median age was 12.9 years (range: 5.7–17.8; mean 12.5 ± 3.0), and the median body weight was 4.4 kg (range: 1.5–16.3; mean 5.0 ± 2.8). Spayed females and castrated males each comprised 46.2% of cases, while intact males and females accounted for 4.6% and 3.1%, respectively. Notably, 69.2% of dogs were classified as ASA grade III or higher; however, this was not significantly associated with postoperative complications. Comorbidities were recorded in 66.2% of patients. In terms of breed distribution, Toy Poodles were most common (35.4%), followed by Maltese (16.9%), Pomeranians (13.8%), and mixed breeds (12.3%) (Table 1).

### 3.2. Preoperative Laboratory Findings

Abnormal serum biochemical values were common before surgery. Liver enzyme activities showed wide variation, with frequent increases in ALT (range, 2–2927 U/L), ALP (range, 0–8089 U/L), and GGT (range, 3–430 U/L), indicating a consistent pattern of hepatobiliary enzyme elevation (Table 2). AST (range, 4–1000 U/L) and total bilirubin (range, 0–7.3 mg/dL) were moderately increased in some dogs, while albumin (range, 1.6–4.1 g/dL) generally remained within normal limits. CRP (range, 9–210 mg/L) was elevated in most cases, suggesting the coexistence of systemic inflammation. Among these analytes, ALP and GGT exhibited the most consistent and marked elevations, often rising in parallel with ALT and AST.

### 3.3. Ultrasound Findings

**Figure 1:**
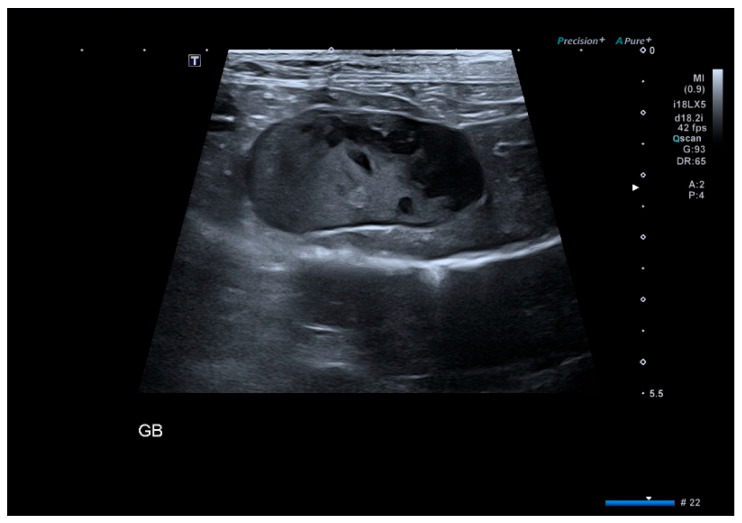
Immobile sludge ultrasonographic pattern of gallbladder mucocele. (Pattern I). Immobile sludge pattern featuring near-complete filling of the gallbladder lumen with homogenous echogenic bile and absent movement or layering. This appearance reflects advanced intraluminal bile organization consistent with gallbladder mucocele. Abbreviations on the ultrasound image indicate system-generated labels: GB, gallbladder; T, transducer orientation marker; “#” and green letters represent default device display parameters.

**Figure 2:**
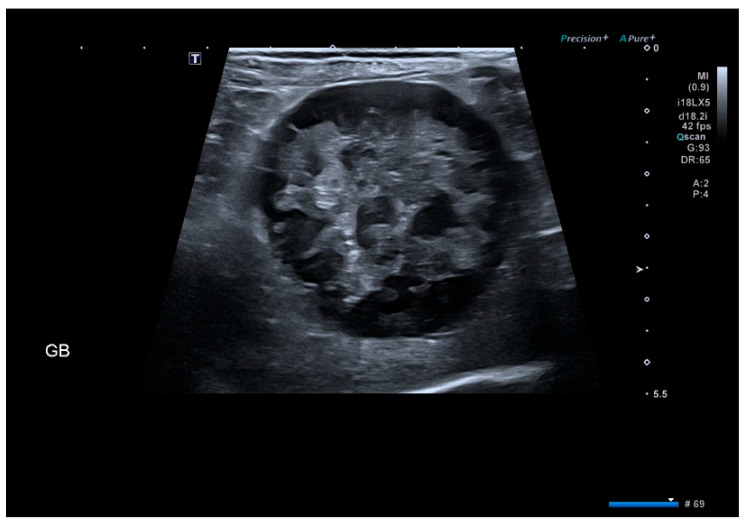
Ultrasonographic appearance of the stellate/striated pattern. (Pattern II). It demonstrates multiple hyperechoic linear and branching striations within inspissated bile. This was the most common morphologic pattern in the study cohort (41.5%).

**Figure 3:**
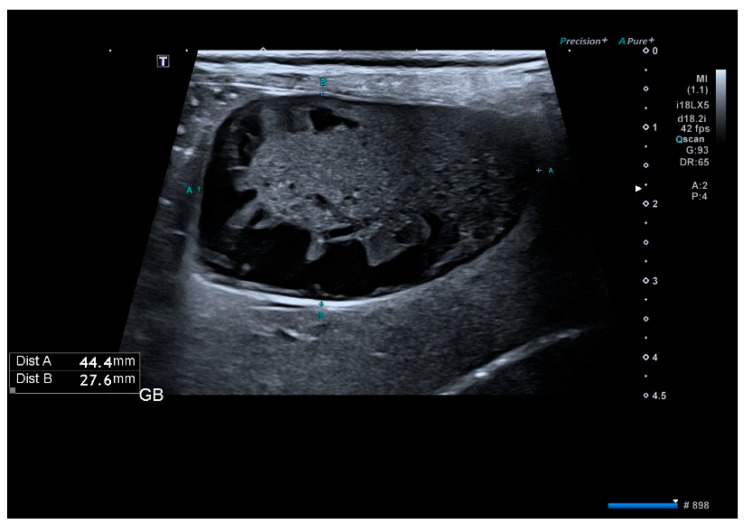
Ultrasonographic image of the gallbladder showing the classic “kiwi fruit” pattern. (Patterns III–IV). It is characterized by a central stellate region of echogenic immobile bile with radiating strands and peripheral anechoic bile. This pattern is indicative of an organized gallbladder mucocele.

Gallbladder mucoceles were identified in the majority of dogs based on ultrasonographic patterns. Among 65 dogs, the stellate/striated pattern was most common (41.5%), followed by the classic “kiwi fruit” appearance (29.2%) and immobile sludge (27.7%). Only one dog (1.5%) showed an unremarkable gallbladder. However, cholecystectomy was performed in this dog due to persistent gastrointestinal signs and progressive elevation of hepatobiliary enzymes. In addition, biliary obstruction—defined as extrahepatic biliary duct dilation or impaired bile outflow on ultrasonography—was identified in 27 dogs (41.5%). These findings confirm that most cholecystectomy cases in this cohort were associated with organized or immobile echogenic bile patterns consistent with gallbladder mucocele, as illustrated in Figure 1, Figure 2 and Figure 3 [3].

### 3.4. Bile Culture and Antimicrobial Resistance

**Figure 4:**
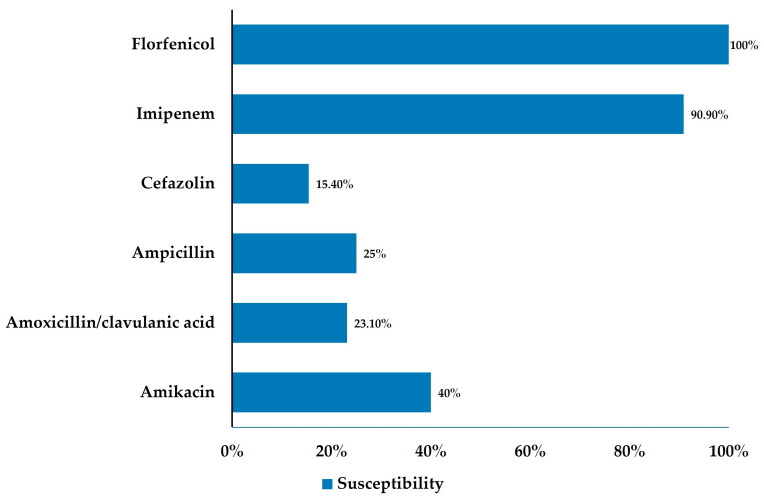
Antimicrobial susceptibility of isolated organisms (culture-positive only, *n* = 13). Florfenicol and imipenem showed the highest susceptibility, while β-lactam antibiotics demonstrated substantially reduced activity against biliary isolates.

Bile cultures were performed in 61 dogs, and 13 (21.3%) yielded positive results (95% CI, 11.3–31.2; *p* < 0.001). Among these, multidrug-resistant (MDR) organisms were identified in 7 dogs (11.5%) and extensively drug-resistant (XDR) organisms in 4 dogs (6.6%). Within the culture-positive group, MDR and XDR isolates accounted for 53.8% and 30.8% of cases, respectively, while pan-susceptible isolates represented only 15.4%. These findings indicate that more than half of the bile-positive samples harbored bacteria with advanced antimicrobial resistance profiles (Table 3). Among the 13 culture-positive cases, the most frequently isolated organism was *Escherichia coli* (*n* = 5, 38.5%), followed by *Enterococcus* spp. (*n* = 4, 30.8%) and *Enterobacter cloacae* (*n* = 2, 15.4%). Other isolates included *Pseudomonas aeruginosa*, *Enterococcus hirae*, and *Enterobacter homaechei* (each *n* = 1, 7.7%). Gram-negative bacteria accounted for the majority of isolates. Mixed bacterial growth, defined as the isolation of more than one bacterial species from bile sample, was observed in 5 of 13 positive cases (38.5%) (Table 3). Antimicrobial susceptibility test showed consistently high sensitivity to imipenem (90.9%) and florfenicol (100.0%), but notably poor susceptibility to commonly used β-lactam antibiotics, including amoxicillin–clavulanate (23.1%) and cefazolin (15.4%). These findings underscore the clinical importance of routine bile culture and susceptibility testing in dogs undergoing cholecystectomy (Figure 4).

### 3.5. Antibiotic Matching and Postoperative Outcomes

Within 30 days after surgery, major complications were documented in 8 of 65 dogs (12.3%), and the 30-day mortality rate was 4.6% (3/65). In total, 23 dogs (35.4%) required postoperative intensive care, and 6 (9.2%) were readmitted within 90 days. There was no significant association between matched antibiotic therapy and the occurrence of major complications (χ^2^ = 1.29, *p* = 0.256; OR = 3.93, 95% CI: 0.31–49.12). Likewise, antibiotic matching did not significantly affect hospital stay duration (rate ratio = 1.13, 95% CI: 0.62–2.06, *p* = 0.700).

### 3.6. Histopathology of Gallbladder and Liver

**Figure 5:**
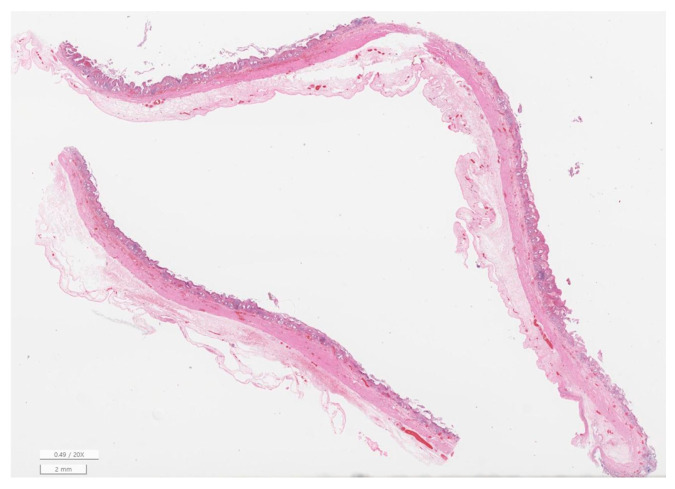
Gallbladder mucosal hyperplasia with inspissated mucus (H&E, ×20). Marked mucosal hyperplasia with deeply folded epithelium and accumulation of inspissated laminated mucus along the luminal surface, consistent with gallbladder mucocele.

**Figure 6:**
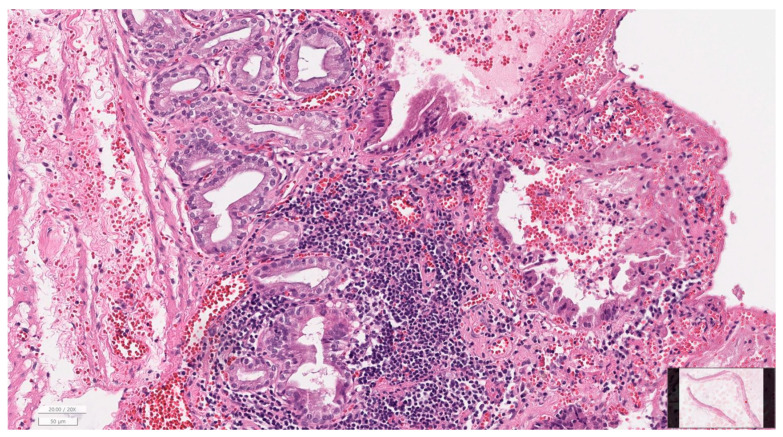
Chronic cholecystitis with submucosal fibrosis (H&E, ×200). Dense lymphoplasmacytic infiltration and moderate submucosal fibrosis are present, accompanied by focal epithelial attenuation, indicating chronic inflammatory changes associated with gallbladder mucocele.

**Figure 7:**
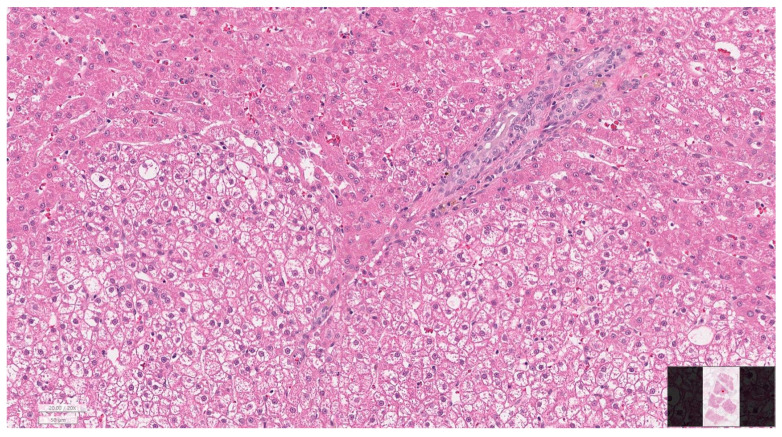
Portal hepatitis with mild cholangiopathy and early periportal fibrosis (H&E, ×200). Mononuclear inflammatory infiltrates are evident within the portal region, together with mild cholangiolar proliferation and early periportal fibrosis, representing reactive hepatic changes secondary to gallbladder disease.

Gallbladder histopathology was available for 52 of 65 cases (80.0%) (Table 4). Mild to moderate inflammation was most frequent (61.5%), with severe inflammation present in 3 dogs (5.8%). The majority of gallbladders showed no fibrotic changes (84.6%), with only a small number displaying mild to moderate fibrosis. Mucosal hyperplasia was a common finding (90.4%). No bacteria were identified histologically, and gallbladder rupture was documented in one case (1.9%). Liver histopathology was available in 21 cases (32.3%) (Table 5). Mild to moderate hepatitis (grades 1–2) was most common (47.6%), while severe hepatitis was present in 3 dogs (14.3%). Cholangiopathy was absent in 38.1% of cases but present at mild (28.6%) or moderate (33.3%) levels in others. Fibrosis ranged from absent (42.9%) to severe (19.0%). Because of the limited number of liver biopsies, statistical evaluation of associations between gallbladder and liver findings was not performed. Representative microscopic findings are presented in Figure 5, Figure 6 and Figure 7, illustrating gallbladder mucosal hyperplasia with inspissated mucus, chronic cholecystitis with submucosal fibrosis, and portal hepatitis with mild cholangiopathy and early periportal fibrosis.

### 3.7. Breed Predisposition: Over-Representation of Toy Poodles

**Figure 8:**
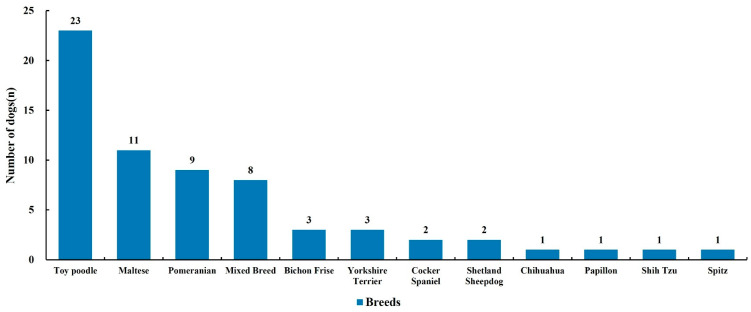
Breed distribution of 65 dogs that underwent cholecystectomy for gallbladder mucocele. Toy Poodles were the most frequently affected breed, followed by Maltese and Pomeranians, whereas other breeds were represented at markedly lower frequencies.

Breed analysis showed that Toy Poodles represented the largest proportion of cases (23/65, 35.4%) (Figure 8). Compared to their prevalence in the hospital population (15.2%), Toy Poodles were significantly over-represented (*p* < 0.001; 95% CI: 23.9–48.2). Toy Poodles had approximately three times higher odds of undergoing cholecystectomy compared with other breeds (OR = 3.1, 95% CI: 1.9–5.1, *p* < 0.001).

## 4. Discussion

The predominance of elderly, small-breed dogs in this study aligns with previous reports of gallbladder mucocele and related biliary disorders [1,20]. Although a large proportion were ASA III or higher, the grade was not significantly linked to complications, consistent with previous studies [7,9].

This may indicate that optimization of anesthetic monitoring and individualized perioperative care can mitigate the risks associated with high ASA status [8]. The bile culture positivity rate observed in this study was consistent with earlier investigations of canine hepatobiliary disease, which reported similar frequencies of positive bile cultures [15,16,17]. Consistent with previous findings, *E. coli* and *Enterococcus* spp. were the most commonly isolated organisms, supporting the concept that most biliary infections originate from enteric bacteria [16,18].

Notably, more than half of the culture-positive samples yielded multidrug-resistant (MDR) or extensively drug-resistant (XDR) organisms, reflecting the increasing concern of antimicrobial resistance in veterinary medicine [18,29,30]. In contrast to earlier studies such as [16], where susceptibility to first-choice β-lactams—particularly amoxicillin–clavulanate—remained moderate to high, our isolates demonstrated substantially lower susceptibility [18]. Recent studies have also reported reduced susceptibility to β-lactams among hepatobiliary isolates from dogs and cats [18,29]. Given this trend and the predominance of Escherichia coli in bile cultures, empirical combination therapy using a β-lactam with a fluoroquinolone may be considered in severe or high-risk cases to broaden coverage until culture results become available, as suggested in previous veterinary antimicrobial studies [31]. Nevertheless, nearly all isolates in our cohort retained sensitivity to imipenem and florfenicol, suggesting that these antimicrobials may remain effective against biliary pathogens exhibiting advanced resistance profiles.

These observations indicate that postoperative outcomes are influenced by multifactorial interactions rather than a single determinant. By integrating microbiologic, clinicopathologic, histopathologic, and epidemiologic findings, this study provides practical information that may help guide clinical decision-making and improve outcomes in dogs undergoing cholecystectomy.

Clinically, bile infection or multidrug-resistant organisms did not translate into increased postoperative morbidity or prolonged hospitalization in this cohort. Early surgical intervention and standardized perioperative antimicrobial therapy likely mitigated the short-term impact of biliary infection on outcomes. Similarly, antibiotic matching showed no significant association with postoperative complications or hospitalization duration, suggesting that perioperative management, surgical timing, and overall patient stability may have a greater influence on short-term outcomes than antibiotic selection alone. The universal use of postoperative broad-spectrum antibiotics in all dogs may have further mitigated the short-term clinical impact of bile infection and antimicrobial resistance, contributing to the lack of association between culture status and postoperative outcomes.

Therefore, while bile culture remains indispensable for guiding targeted antimicrobial therapy, our findings indicate that a positive bile culture result alone should not delay cholecystectomy when clinical and imaging criteria support surgical intervention.

Histopathologic examination confirmed frequent mucosal hyperplasia in the gallbladder and variable inflammatory and fibrotic changes in the liver. It is also important to note that positive bile cultures do not necessarily correspond to histopathologic evidence of inflammation or tissue injury, because infection may be focal, transient, partially controlled by prior antimicrobial therapy, or confined to bile without extension into adjacent tissue; however, the limited number of biopsy samples precluded further statistical evaluation.

The biochemical patterns observed—characterized by cholestatic rather than hepatocellular enzyme elevation—are consistent with previous reports describing similar trends in dogs with gallbladder disease. Mild to moderate hyperbilirubinemia, often indicating partial biliary obstruction, has likewise been reported in dogs with gallbladder mucocele or chronic cholecystitis [4,5,6]. The results suggest that gallbladder disease in dogs may arise from a combination of factors, including cholestasis, reactive hepatic changes, and systemic inflammation. Beyond microbial and biochemical findings, the strong breed predisposition observed in Toy Poodles suggests that clinicians should maintain heightened awareness of gallbladder disease in this population and consider earlier diagnostic screening [11,20].

This study has several limitations. Its retrospective, single-center design introduces potential selection and information bias. Second, the number of liver biopsy samples was limited, preventing meaningful evaluation of associations between gallbladder and hepatic pathology [10]. Analyses of antibiotic matching and complications risk may have been underpowered due to the modest number of culture-positive cases. Furthermore, long-term outcomes, including survival beyond 30 days and recurrence, could not be comprehensively assessed [9]. Additionally, anaerobic bacterial culture was only performed in a limited number of cases and was not included in the final analysis, which may have led to underestimation of the true prevalence of biliary infection or mixed infections involving anaerobes. The type of inflammatory infiltrate could not be evaluated because it was not consistently specified in the pathology reports, and thus its relevance to bile culture status remains undetermined.

Future studies should include larger, multi-institutional cohorts to improve statistical power and generalizability. Prospective research is warranted to determine the long-term prognostic implications of bile culture positivity and antimicrobial resistance [29]. Expanded histopathological evaluation of both gallbladder and liver tissue may clarify the interplay between these organs in biliary disease [10].

The demographic characteristics of this cohort are also consistent with the established epidemiology of gallbladder mucocele [2,3,12,13]. GBM predominantly affects geriatric, small-breed dogs, and the high proportion of elderly and toy-breed dogs in this study reflects this trend [3,12,13]. Multiple risk factors have been implicated in the development of GBM, including hyperadrenocorticism, hypothyroidism, and hyperlipidemia, which may contribute to impaired gallbladder motility and excessive mucin accumulation [2,11]. The high comorbidity rate observed in the present cohort therefore supports the multifactorial nature of the disorder. In addition, chronic systemic inflammation and cholestatic hepatic injury have been proposed to exacerbate epithelial damage and accelerate progression to clinically significant disease [4,5]. Taken together, these findings suggest that GBM does not arise from a single pathogenic mechanism but rather from the interaction of age-related metabolic disease, biliary dysmotility, and systemic inflammatory processes [10,14].

In our study, Toy Poodles exhibited the highest breed-associated risk for gallbladder mucocele, suggesting a stronger predisposition compared with previous reports, in which Poodles were described merely as one of several frequently affected small breeds [11,12,20]. Similar findings have also been observed in Asian and Korean cohorts, where Toy Poodles were among the breeds most frequently affected by gallbladder mucocele or those undergoing cholecystectomy [3,11].

Finally, exploring genetic and epidemiologic factors in predisposed breeds, particularly Toy Poodles, may provide important insights into the pathogenesis of gallbladder disease and help develop better preventive strategies in these breeds. This pattern mirrors prior reports that identified breed overrepresentation relative to hospital caseloads [14] and elevated odds for specific breeds in case–control studies [20].

Further application of genome-wide association studies (GWASs) in Toy Poodles could reveal genetic variants that increase susceptibility to this condition. Although GWASs have not yet been conducted in this breed, previous work—identifying an ABCB4 insertion mutation associated with gallbladder mucocele formation in dogs [21] and breed-predisposition analyses demonstrating overrepresentation of certain breeds [12,13] —supports the potential value of this approach for uncovering hereditary risk factors.

## 5. Conclusions

This study assessed the clinical relevance of bile culture, antimicrobial susceptibility testing, and hepatobiliary histopathology in 65 dogs undergoing cholecystectomy. Although the bile culture positivity rate was modest, more than half of the positive samples contained multidrug-resistant or extensively drug-resistant organisms, underscoring the importance of routine culture and susceptibility testing to guide appropriate antimicrobial therapy. Gallbladder and hepatic histopathology frequently revealed chronic inflammatory and fibrotic changes, and Toy Poodles were overrepresented among affected dogs, suggesting a possible breed predisposition. Overall, these findings emphasize the diagnostic value of integrating microbiologic and histopathologic evaluation with culture-based antimicrobial selection to optimize perioperative management of dogs undergoing cholecystectomy.

## Figures and Tables

**Table 1:** Baseline characteristics of 65 dogs undergoing cholecystectomy.

Characteristic	*n* (%)
Age, years	12.5 ± 3.0 (median 12.9)
Body weight, kg	5.0 ± 2.8 (median 4.4)
Sex	SF 30 (46.2), MC 30 (46.2), M 3 (4.6), F 2 (3.1)
Comorbidity	Present 43 (66.2), Absent 22 (33.8)
High-risk ASA grade (≥III)	45 (69.2)
Breed	Toy poodle 23 (35.4), Maltese 11 (16.9), Pomeranian 9 (13.8), Mixed 8 (12.3), Others 14 (21.5)

Values are presented as mean ± SD (Standard Deviation). ASA: American Society of Anesthesiologists physical status classification. Breeds with fewer than three cases were grouped as “Others.” SF, spayed female; MC, castrated male; M, intact male; F; intact female.

**Table 2:** Preoperative laboratory findings in dogs undergoing cholecystectomy.

Parameter	Reference Range	Mean ± SD	Median	Above Ref (%)
ALT (U/L)	19–70	489.5 ± 617.8	167.7	72.3
ALP (U/L)	15–127	1022.6 ± 1485.6	454.9	72.3
GGT (U/L)	0–6	53.9 ± 70.1	24.6	87.1
AST (U/L)	15–43	127.0 ± 199.2	38.0	48.3
T-bilirubin (mg/dL)	0–0.4	1.17 ± 1.82	0.2	33.8
Albumin (g/dL)	2.2–3.9	2.86 ± 0.49	2.86	7.8
WBC (×10^3^/µL)	5.32–16.92	14.28 ± 7.74	13.5	32.3
CRP (mg/L)	0–9	76.1 ± 71.0	49	73.8

Reference intervals were based on manufacturer-provided ranges for canine samples established by each analyzer (DRI-CHEM NX700, Fujifilm; BS-430, Mindray; BC-60R Vet, Mindray). Values are presented as mean ± SD (standard deviation) and median, with the percentage of dogs above the reference interval indicated. ALT: alanine aminotransferase; ALP: alkaline phosphatase; GGT: γ-glutamyl transferase; AST: aspartate aminotransferase; WBC: white blood cell count; CRP: C-reactive protein; T-bilirubin: total bilirubin.

**Table 3:** Bile culture positivity and resistance patterns (*n* = 61).

Variable	Category	*n* (%)	95% CI	*p*-Value
Bile culture	Positive	13/61 (21.3)	11.3–31.2	<0.001
	Negative (no growth)	48/61 (78.7)	66.9–87.1	<0.001
Resistance classification	MDR	7/61 (11.5)	3.2–18.7	0.032
	XDR	4/61 (6.6)	2.0–12.5	<0.001
	Pan-susceptible	2/61 (3.3)	0.4–6.8	<0.001

MDR (multidrug-resistant): non-susceptibility to at least one agent in ≥3 antimicrobial categories. XDR (extensively drug-resistant): non-susceptibility to all but ≤2 categories. Pan-susceptible: susceptible to all tested antimicrobial categories. Percentages are based on dogs with available culture results (*n* = 61). *p*-values < 0.05 were considered statistically significant.

**Table 4:** Histopathological findings of the gallbladder (*n* = 52 *).

Variable	Category	*n* (%)
Inflammation	None	17 (32.7)
	Mild	22 (42.3)
	Moderate	10 (19.2)
	Severe	3 (5.8)
Fibrosis	None	44 (84.6)
	Mild	7 (13.5)
	Moderate	1 (1.9)
Mucosal hyperplasia	Present	47 (90.4)
Bacteria on histology	Present	0 (0.0)
Perforation	Present	1 (1.9)

* Gallbladder histopathology was evaluated in 52 dogs undergoing cholecystectomy. Specimens were graded based on the severity of mucosal hyperplasia, inflammation, and fibrosis. Mucinous material, cholesterol clefts, and epithelial sloughing were recorded when present.

**Table 5:** Histopathological findings of the liver (*n* = 21 *).

Variable	Category	*n* (%)
Hepatitis grade	None	8 (38.1)
	Mild	3 (14.3)
	Moderate	7 (33.3)
	Severe	3 (14.3)
Cholangiopathy grade	None	8 (38.1)
	Mild	6 (28.6)
	Moderate	7 (33.3)
Fibrosis stage	None	9 (42.9)
	Mild	3 (14.3)
	Moderate	5 (23.8)
	Severe	4 (19.0)

* Percentages calculated after excluding missing data (21/65 cases available). Lesions were categorized according to the presence and severity of inflammation, fibrosis, and cholestasis.

## Data Availability

The data presented in this study are available on reasonable request from the corresponding author. The data are not publicly available due to privacy restrictions involving client-owned animals treated at Helix Animal Medical Center.

## Abbreviations

The following abbreviations are used in this manuscript:

ALT	Alanine aminotransferase
ALP	Alkaline phosphatase
AST	Aspartate aminotransferase
GGT	Gamma-glutamyl transferase
CRP	C-reactive protein
T-bil	Total bilirubin
ASA	American Society of Anesthesiologists
GBM	Gallbladder mucocele
MDR	Multidrug-resistant
XDR	Extensively drug-resistant
AMR	Antimicrobial resistance
AST (test)	Antimicrobial susceptibility testing
EUCAST	European Committee on Antimicrobial Susceptibility Testing
CLSI	Clinical and Laboratory Standards Institute
IACUC	Institutional Animal Care and Use Committee
LOS	Length of stay
SPSS	Statistical Package for the Social Sciences
